# PTEN suppresses the inflammation, viability, and motility of AP-AR42J cells by activating the Wnt/β-catenin pathway

**DOI:** 10.1039/c8ra08998a

**Published:** 2019-02-13

**Authors:** Hongtao Yan, Li Jiang, Hong Zou, Tao Chen, Hongyin Liang, Lijun Tang

**Affiliations:** General Surgery Center of PLA, General Hospital of Western Theater Command No. 270 Rong Du Road, Jinniu District Chengdu Sichuan Province 610083 P. R. China lijuntang1@aliyun.com +86-028-86570326; Cardiac Care Unit of Cardiothoracic Surgery, General Hospital of Western Theater Command Chengdu Sichuan 610083 P. R. China

## Abstract

Acute pancreatitis (AP), a kind of common acute abdominal disease and typical chemical inflammation, is commonly caused by pancreatin digestion of the pancreas and surrounding tissues. The gene for phosphate and tension homology deleted on chromosome ten (PTEN) is a tumor suppressor that regulates numerous cellular processes. In the present study, we have elaborately investigated the effect of PTEN on the alleviating of AP and its underlying mechanisms. Firstly, we demonstrated an up-regulation of PTEN in the pancreatic tissues from AP rats by immunochemistry, qRT-PCR and western-blot assays. Subsequently, cellular experiments exhibited that PTEN has a significant inhibition effect on the proliferation, invasion and migration of AP cells. Further underlying mechanism studies showed that the growth of AP cells was mainly restrained by PTEN in the G1 phase through activation of the Wnt/β-catenin pathway, which can be demonstrated by the down-regulation of various pro-inflammatory cytokines such as IL-6, IL-10, TNF and IL-1β. Taking these results together, we can draw the conclusion that PTEN plays a significant role in suppressing the inflammation, viability and motility of acute pancreatitis and could be a potential target for AP therapies.

## Abstract 2

Acute pancreatitis (AP), a kind of common acute abdominal disease and typical chemical inflammation, is commonly caused by pancreatin digestion of the pancreas and surrounding tissues.

## Graphical abstract

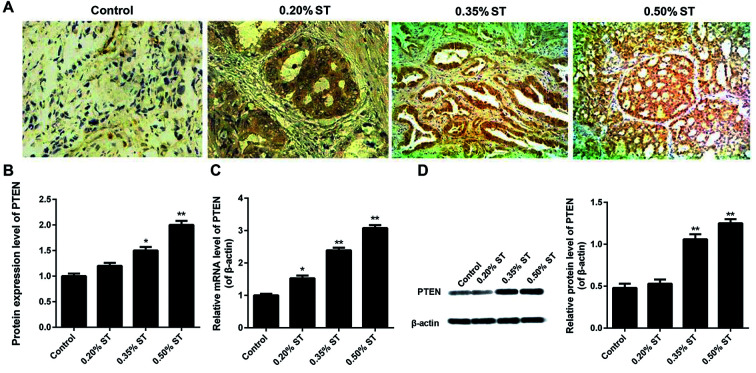

## Introduction

Acute pancreatitis (AP), a kind of common acute abdominal disease, is one of the representative chemical inflammations caused by the pancreatin digestion of the pancreas and surrounding tissues.^1^ Generally, AP is composed of two types including acute edematous pancreatitis (AEP) and acute hemorrhagic necrotizing pancreatitis (AHNP). The AEP, characterized by pancreatic edema, is more commonly seen than AHNP and accounts for more than 90% of AP.^2^ Although the exact pathogenesis of AP remains ill-defined, the over-activation of pancreatic enzymes and inflammatory pathways activation have been demonstrated to be the critical regulators of the pancreas's self-digestion.^3^ Previous reports have demonstrated that impaired autophagy, and imbalance of cathepsin expression are the main causes leading to zymogen activation while the activation of NFκB easily results in inflammation.^4^ Even though acute pancreatitis represents the leading cause of significant morbidity and mortality, an efficient treatment strategy is still needed urgently.^5^

Wnts, together with their downstream effectors, are one of the most important modulators, which have a remarkable effect on the multifarious biological functions of cells such as cell senescence, death, differentiation, and metastasis.^6,7^ There are usually two types of Wnt pathways: the classical β-catenin-dependent pathway (Wnt/β-catenin pathway) and the non-classical β-catenin-independent pathway.^8^ Previous reports have demonstrated that the Wnt/β-catenin pathway plays a hostile role in the migration and invasion of multiple carcinoma cells.^9–11^ Additionally, studies recently put forward a close relation of the Wnt signaling pathway and pancreas-related diseases based on the fact that dysregulation of the Wnt pathway was found to be related to the specification, proliferation, differentiation and function of pancreatic organogenesis.^12^ Based on this, regulation of Wnt/β-catenin became a research hotspot and was supposed to hold great potential in alleviating acute pancreatitis.^12^

The phosphatase and tension homolog gene (PTEN), a namely tumor suppressor gene located on chromosome 10q23.31, has been regarded as one of the most commonly mutated tumor suppressors.^13^ Previous studies have reported that PTEN has a significant effect on the suppression of the migration and invasion of various cancer cells.^14–17^ In contrast, germline mutations of PTEN or the complete loss of PTEN protein expression are significantly associated with autosomal dominant syndromes, neurological deficits and an increased risk of cancer.^13^ The underlying mechanism of the PTEN inhibition of cancer mainly lies in the fact that it can regulate numerous cellular processes such as survival, proliferation, cellular architecture, and energy metabolism.^18^

The regulation of cellular processes by PTEN is realized by the effects of various signaling pathways in cells.^19,20^ Among those, the Wnt/β-catenin pathway represents one the most important PTEN downstream targets. However, there are very few studies related to the effect of PTEN on the migration and invasion of AP cells to date. In the present study, we aimed to probe the potential effects of PTEN on the migration and invasion of AP on rat models and human AP acinar cell models. For animal experiments, the retrograde bile duct injection method was introduced to establish the AP rat models.^21^ Meanwhile, AP acinar cell models were built by the caerulein inducing approach.^22^ Subsequently, suppressing the effect of PTEN on the inflammation, viability and motility of AP were elaborately investigated. Besides, the correlation between PTEN and the Wnt/β-catenin pathway in AP cells were further determined in our study.

## Materials and methods

### Ethical approval

Healthy adult male Sprague-Dawley (SD) rats (weighing 350.0–400.0 g, aged 9–10 weeks) were obtained from the Laboratory Animal Center of Sichuan province. All animal procedures in the present study were performed in accordance with the Guidelines for Care and Use of Laboratory Animals of Sichuan University and experiments were approved by the Animal Ethics Committee of West China School of Pharmacy.

### Establishment of the AP animal model

The AP rat models were established using a previous method with a slight modification.^21^ In brief, the rats were abdominally anesthetized by 10% chloral hydrate (XiLong Scientific, Shenzhen, Guangdong, China). After the rats were immobilized by a fixed device, a slit was incised in the middle of abdomen to expose the duodenum and pancreaticobiliary duct. Subsequently, the pancreatic duct near the porta hepatis was clamped using a non-invasive vessel clamp, followed by a retrograde puncture through the duodenal and duodenal papillary by a scalp needle with a diameter of 0.45 mm. For the induction of acute pancreatitis, 0.20%, 0.35%, and 0.50% synthetic caerulein (0.1 mL/100 g; XiLong Scientific) was respectively injected into the pancreaticobiliary duct, at a constant rate of 0.1 mL min^−1^. Rats injected with saline alone were used as the control.

### Cell model, genes, and plasmids

Human pancreatic acinar AR42J cell lines were obtained from the Cell Bank of Chinese Academy of Sciences and incubated in Ham's F-12K medium (F-12K; Gibco, Carlsbad, CA, USA) supplemented with 10% fetal bovine serum (FBS, Gibco), 100 U mL^−1^ penicillin, and 0.1 mg mL^−1^ streptomycin. To obtain the AP cell models, AR42J cells were seeded in 6-well plates at a density of 1 × 10^7^ cells per mL. Twenty-four hours later, 10 nmol L^−1^ caerulein was added into each well and incubated with cells for another 24 h. Finally, the obtained AP cells (AP-AR42J) were transfected with various genes including scramble-shRNA, PTEN, PTEN shRNA, and pCDH empty vector (Invitrogen, Carlsbad, CA, USA).

### Grouping

For the animal experiments, thirty-two rats were randomly grouped (*n* = 8) and treated as follows: (1) control group: rats injected with saline alone; (2) 0.20% ST group: rats injected with 0.20% sodium taurocholate solution; (3) 0.35% ST group: rats administrated with 0.35% sodium taurocholate solution; (4) 0.50% ST group: rats treated by 0.50% sodium taurocholate solution. For the cell experiments, the AR42J cells were induced with caerulein followed by grouping as follows: (1) scramble-shRNA group: AP-AR42J cells transfected with scramble-shRNA; (2) PTEN-shRNA group: AP-AR42J cells transfected with PTEN-shRNA; (3) pCDH group: AP-AR42J cells transfected with pCDH; (4) PTEN overexpression group: AP-AR42J cells transfected with PTEN.

### Immunohistochemistry

All the rats in each group were sacrificed and the pancreas tissues of the AP rats and the control ones were collected. For the tissue fixation, the obtained pancreas tissues were immersed in 4% paraformaldehyde for 12 h followed by dehydration using 30% sucrose solution. Then slices of the pancreas tissues (14 μm) were prepared and co-incubated with primary antibodies against PTEN (dilution, 1 : 50; Abcam, ab170941; rabbit anti-rat) overnight. Subsequently, horseradish peroxidase-conjugated goat anti-rabbit IgGs (dilution, 1 : 1000; Abcam, ab150077) was introduced and incubated with the slices for 1 h. Finally, the slices were treated with diaminobenzidine (DAB, Sigma-Aldrich, St. Louis, MO, USA) before determination under a microscope (OLYMPUS, Tokyo, Japan).

### Cell viability analysis

To evaluate the anti-proliferation ability of PTEN on AP-AR42J cells, a 3-(4,5-dimethylthiazol-2-yl)-2,5-diphenyltetrazolium bromide (MTT; AMERCO, USA) assay was performed. Briefly, cells in the logarithmic phase were seeded in each well of the 96-well plates at a density of 6 × 10^3^ cells. After incubation for different times (24, 48, or 72 h), 10 μL of MTT (5 mg mL^−1^) was added and incubated with cells for 4 h to form the formazan. Thereafter, 150 μL of dimethyl sulfoxide (DMSO; XiLong Scientific) was supplemented and further incubation for 10 min was allowed. Finally, the absorbance was detected at 490 nm wavelength using a microplate reader (BIO-RAD, California, USA) and the cell viability and inhibition rate were evaluated by calculating the percentage of cell survival compared with the control.

### Cell cycle analysis

AP-AR42J cells were seeded in 6-well plates at a density of 1 × 10^7^ cells per well and allowed to grow for 24 h. Then 1 mL of pre-cooling ethanol (70%) was added and incubated with cells at 4 °C. After overnight incubation, 500 μL of PBS containing PI (50 μg mL^−1^), RNase A (100 μg mL^−1^), or Triton X-100 (0.2%) were added, respectively. After 30 min of incubation under dark conditions, the cell cycles were measured *via* the help of flow cytometry (BD Biosciences, San Diego, CA, USA).

### Wound-healing assay

To investigate the wound-healing ability of the AP-AR42J cells, various gene transfected cells were seeded in 24-well plates, respectively, at a density of 1 × 10^5^ cells per mL. After the cells had grown to 100% of confluence, horizontal lines were drawn at the bottom of each well using a 10 μL pipette tip. Subsequently, all of the cells were washed three times by Hanks solution (Gibco) to remove the unattached cells and supplemented with fresh serum-free medium (Gibco). Then qualitative images were taken by an optical microscope at 0, 24, and 48 h, respectively.

### Transwell assay

Given the significant role of the transwell assay in providing a relatively simple *in vitro* approach to evaluate cell invasion, we therefore applied such a method to investigate the invasion ability of the AP-AR-42J cells. The cells (5 × 10^5^) pre-transfected with various genes were located on the upper chamber, which we pre-coated with Matrigel (BD Bioscience), while the lower ones were filled only with F-12K medium containing 10% FBS. After 24 h of incubation, cells migrated from the upper chamber to the lower ones and were fixed by 4% paraformaldehyde and stained with 0.5% crystal violet (Solarbio, Beijing, China). Meanwhile, the non-migrated cells were discarded along with the upper chamber without any further processing. Finally, the crystal violets in cells were subjected to qualitative and quantitative statistics under a microscope.

### Western-blot analysis

After segregation by 12% sodium dodecyl sulfate polyacrylamide gel electrophoresis (SDS-PAGE), the protein lysates of the tissue samples or the cultured cells were transferred to a PVDF membrane (Millipore, Billerica, MA, USA). Then the membrane, which was transferred with various proteins, was allowed to interact with different primary anti-bodies, including anti-PTEN, -Wnt3a, -β-catenin, -TCF-4, -c-Myc, -cyclin D1, and -β-actin. After overnight incubation, horseradish peroxidase conjugated secondary antibodies (dilution, 1 : 5000, Abcam, ab205718, goat antirabbit) were applied and incubated with the membranes under room temperature for 1 h. Finally, the results were observed using an ECL system (Amersham Pharmacia, Piscataway, NJ, USA).

### Real-time reverse transcription PCR (qRT-PCR) analysis

The total RNA was extracted from the tissue samples or cultured cells by the TRIzol reagent (Thermo Fisher, New York, USA) followed by reverse transcribing to cDNA using the Reverse Transcription Kit (Sigma, Munich, Germany). After amplification, the comparative *C*_t_ (threshold cycle) method with arithmetic formulae (2^−ΔΔ*C*_t_^) was applied to determine the relative quantitation of gene expression. PCR cycles were as follows: pretreatment for 10 min at 95 °C, 96 °C for 15 s, 64 °C for 45 s (45 cycles), 96 °C for 15 s, 64 °C for 1 min, 95 °C for 15 s, a final extension at 75 °C for 10 min and held at 4 °C. The primer sequences used in the present study were as follows: PTEN (238 bp for rat and 232 bp for human) forward, ACCCACCACAGCTAGAACTT and reverse, CGCCTCTGACTGGGAATAGT; Wnt3a (245 bp) forward, TGTTGGGCCACAGTATTCCT and reverse, ACTCCCTGGTAGCTTTGTCC; β-catenin (177 bp) forward, GGAGGAGATGTACATTCAGCAGA and reverse, GCCATCACCACGTCCTCT; TCF-4 (249 bp) forward, ATCCTTCCTCCAAACCAGCA and reverse, GGAAAGTGGACATCGGAGGA; c-Myc (217 bp) forward, ATTCTCTGCTCTCCTCGACG and reverse, CTGTGAGGAGGTTTGCTGTG; cyclin D1 (219 bp) forward, CCCTCGGTGTCCTACTTCAA and reverse, CTTAGAGGCCACGAACATGC; β-actin (rat, 247 bp) forward, GGCATCCTGACCCTGAAGTA and reverse, AGGCATACAGGGACAACACA; β-actin (human, 194 bp) forward, GTTACAGGAAGTCCCTCACCC and reverse, CAGACCTGGGCCATTCAGAAA. The β-actin was used as the control.

### Statistical analysis

All the measurement data were presented as mean ± SD and analyzed by the Kruskal-Wallis and Tukey's test using SPSS 15.0 software (SPSS, Chicago, IL, USA). The comparisons among the multiple groups were conducted by the one-way analysis of variance (ANOVA), while the comparisons between the two groups with variance homogeneity were analyzed by means of a *t*-test. All the experiments were conducted repeatedly three times. *P* < 0.05 was considered as a statistical significance.

## Results

### PTEN expression was up-regulated in pancreatic tissues from AP rats

**Fig. 1:**
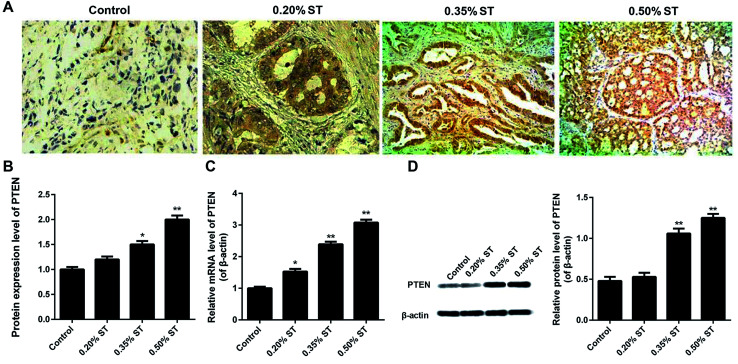
Detection of PTEN in the pancreatic tissue sections after the AP rats were established by retrograde bile duct injection with saline (0.1 mL/100 g) supplemented with 0.20%, 0.35%, and 0.50% sodium taurocholate solution (0.1 mL/100 g; XiLong Scientific), respectively. Immunohistochemical analysis of the pancreatic tissues displayed by qualitative images (A) and quantitative results (B). Furthermore, qRT-PCR (C), and a western-blot (D) assay were performed, respectively, to evaluate the expression levels of PTEN in the pancreatic tissue samples of AP rat models. All the experiments were conducted repeatedly three times. *P < 0.05 and **P < 0.01 *versus* control.

The expression of PTEN in pancreatic tissues was verified by the immunohistochemistry, qRT-PCR assay and western-blot experiments. Fig. 1A and B demonstrate that the levels of PTEN in the pancreatic tissues of AP rats were obviously higher than that of normal rats. Moreover, expression of PTEN in AP rats displayed a dose-dependent manner since increasing the dosage of the sodium taurocholate solution (ST) led to an elevation of PTEN levels. Such results were further confirmed by the qRT-PCR assay. As shown in Fig. 1C, the levels of PTEN in AP rats were nearly 1.6 (0.20% ST group), 2.4 (0.35% ST group), and 3.2 (0.50% ST group) fold of that in normal rats. In addition, qualitative and quantitative analysis of the western-blot experiments indicated a similar trend of PTEN expression to the qRTPCR results (Fig. 1D). These results together suggest that the expression of PTEN was exactly up-regulated in the pancreatic tissues of AP model rats and displayed a ST dose-dependent manner.

### Interference and overexpression of PTEN in caerulein-induced AR42J cells

**Fig. 2:**
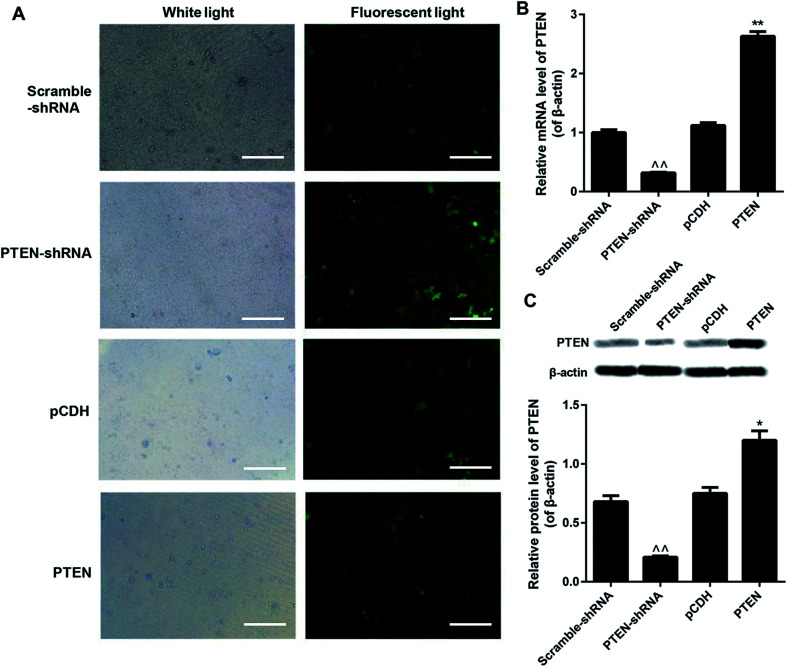
Interference and overexpression of PTEN in caerulein-induced AR42J cells after the caerulein-induced AR42J cells were transfected with fluorescent dye labeled scramble-shRNA, PTEN-shRNA, pCDH, and PTEN RNA, respectively. (A) Green fluorescence observed under the fluorescence microscope displayed that the PTEN-shRNA treated cells exhibited the strongest signal. The bar represents 200 μm. qRT-PCR assay (B) and western-blot experiments (C) further confirming the highest levels of PTEN in the AP cells could be significantly down-regulated by PTEN-shRNA. The assays mentioned here were repeatedly performed at least three times. ^^*P* < 0.01 *versus* scramble-shRNA; *P < 0.05 and **P < 0.01 *versus* pCDH.

To evaluate the knockdown efficiency after gene transfection, all the scramble-shRNA, PTEN, PTEN shRNA, and pCDH empty vectors were labeled with FITC for visualization. Then the green fluorescent signal can be determined under a fluorescence microscope. As shown in Fig. 2A, AP-AR42J cells transfected with PTEN displayed the weakest fluorescent intensity, while cells treated by PTEN-shRNA showed the strongest fluorescent signal. For the scramble-shRNA and pCDH treated cells, no significant differences were observed between the two groups. We speculated that these results were mainly due to the high levels of PTEN in the AP-AR42J cells, which could specifically bind to scramble-shRNA while not other genes. For verification, qRT-PCR experiments and western-blot assays were further conducted. As shown in Fig. 2B and C, the expression level of PTEN in AP-AR42J cells transfected with PTEN-shRNA was remarkably reduced with a knockdown efficiency of about 70%. However, such gene expression in the cells treated by PTEN was significantly elevated while a similar level of PTEN was observed between the scramble-shRNA and pCDH.

### Overexpression of PTEN inhibited the cell viability of caerulein-induced AR42J cells

**Fig. 3:**
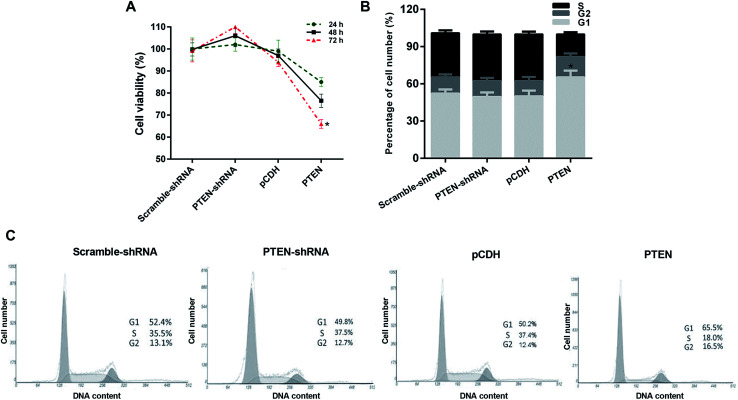
Evaluation of the effect on AP cell growth inhibition and the underlying mechanisms by MTT assay and cell cycle determination, respectively. (A) Results of the MTT assay showed that the AP-AR42J cells transfected with PTEN displayed the lowest cell viability, indicating that overexpression of PTEN could inhibit the proliferation of AP-AR42J cells. (B) Quantitative analysis of the ability of PTEN inhibited proliferation of AP-AR42J cells. (C) Cell cycles of AP-AR42J cells post transfected with various genes (including scramble-shRNA, PTEN-shRNA, pCDH, and PTEN RNA) confirmed that PTEN inhibited the growth of APAR42J cells mainly by blocking the G1 phase of cells. The experiments above were repeated three times. *P < 0.05 *versus* pCDH.

Proliferation of AP-AR42J cells with or without the treatment of PTEN was evaluated by the MTT assay. As shown in Fig. 3A, AP-AR42J cells transfected with PTEN-shRNA displayed the highest cell viability compared with others at any time points. In contrast, after the AP-AR42J cells were transfected with PTEN, the cell viability was significantly decreased, indicating that overexpression of PTEN could inhibit the proliferation of AP-AR42J cells.

### The cell cycle of caerulein-induced AR42J cells was blocked by PTEN overexpression in the G1 phase

In order to investigate the mechanism of PTFN-induced anti-proliferation of APAR42J cells, the cell cycle assay was subsequently determined. As clearly demonstrated in Fig. 3B and C, the percentage of cell number in the G1 phase of APAR42J cells transfected with scramble-shRNA and pCDH were 52.4% and 50.2%, respectively. Simultaneously, for the S phase of cells, the proportion was 35.5% for scramble-shRNA and 37.4% for pCDH. After transfecting with PTEN, the percentage of cell number in the G1 phase and S phase was increased to 65.5% and down-regulated to 18.0%, respectively. However, in contrast to the cells treated by PTEN, cells incubated with PTEN-shRNA displayed that the cell number in the G1 phase and S phase of AP-AR42J cells were reduced to 49.8% and elevated to 37.5%, respectively. Taking these results together, we can draw the conclusion that PTEN inhibited the growth of AP-AR42J cells mainly by blocking the G1 phase of cells.

### Overexpression of PTEN suppressed the migration and invasion of caerulein-induced AR42J cells

**Fig. 4:**
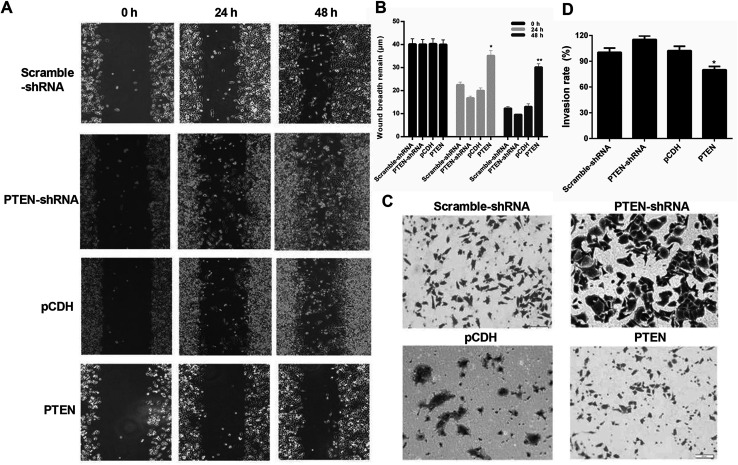
The effect of PTEN on the suppression of the migration and invasion of caerulein-induced AR42J cells was determined by wound-healing and transwell assays post transfecting the cells with various genes. (A) Bright field images of AP-AR42J cells are exhibited, suggesting incompetent wound-healing of AP-AR42J cells transfected by PTEN while the cells treated by PTEN-shRNA exhibited the strongest migration capacity. Semi-quantitative analysis (B) further confirmed these results. The invasion ability of AP-AR42J cells examined by the transwell experiment (C and D) showed that the area of crystal violet induced by cell invasion was significantly decreased by treating with PTEN compared with that treated by reference genes: scramble-shRNA and pCDH. Experiments above were repeated three times. *P < 0.05 *versus* pCDH.

To evaluate the effect of PTEN on suppressing the migration and invasion of AP-AR42J cells, a wound-healing assay was first performed. Fig. 4A and B demonstrated that cells treated by PTEN-shRNA exhibited the strongest migration capacity compared with other groups. The scramble-shRNA and pCDH transfected cells displayed similar migration behavior and were evidently higher than the PTEN-induced cells, suggesting the incompetent wound-healing of AP-AR42J cells transfected by PTEN. Subsequently, transwell experiments were performed. As demonstrated in Fig. 4C and D, the cells subjected to PTEN gene knockout showed the greatest invasion ability since they displayed the most amount of crystal violet compared with the others. However, the area of crystal violet induced by the cell invasion was significantly decreased by treating with PTEN compared with that treated by reference genes: scramble-shRNA and pCDH.

### Overexpression of PTEN affected the Wnt/β-catenin pathway

**Fig. 5:**
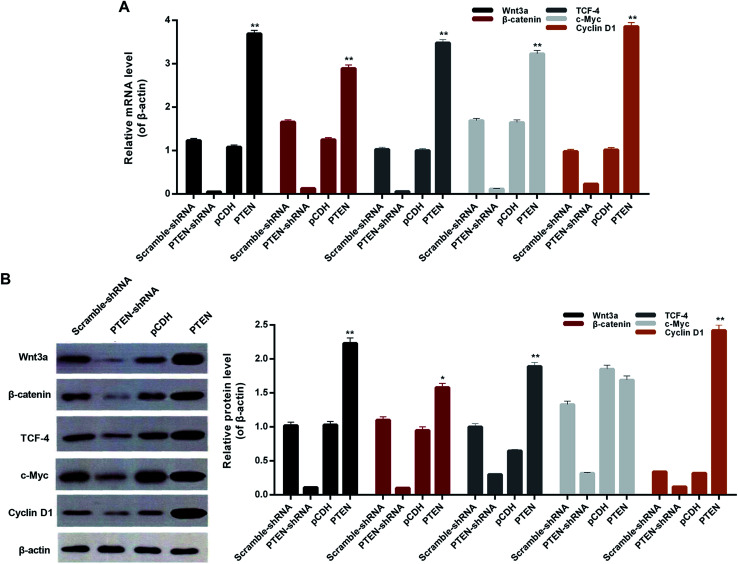
The effect of PTEN on alleviating AP inflammation was proved by detection of downstream genes of the Wnt/β-catenin pathway, including Wnt3a, β-catenin, TCF-4, cMyc, and cyclin D1. (A) qRT-PCR and western-blot assay (B) exhibited that the cells treated by PTEN displayed significant lower levels of inflammatory factors compared with the scramble-shRNA group and pCDH treated ones. However, after the PTEN genes in the AP model cells were knocked down by the corresponding shRNA, the expression levels of IL-6, IL-10, TNF and IL-1β were remarkably elevated. Importantly, all the experiments were conducted repeatedly three times. *P < 0.05 and **P < 0.01 *versus* pCDH.

To verify whether the PTEN disabled the migration and invasion of AP-AR42J cells through affecting the Wnt/β-catenin pathway, the qRT-PCR assay was carried out. As shown in Fig. 5A, the genes of the Wnt/β-catenin pathway including Wnt3a, β-catenin, tTCF-4, c-Myc, and cyclin D1 displayed the lowest levels in the cells transfected with PTEN-shRNA, indicating that the Wnt/β-catenin pathway was remarkably suppressed. However, the expressions of those genes in the AP-AR42J cells could be significantly elevated by treatment with PTEN. These results were further confirmed by the western-blot experiments. As shown in Fig. 5B, the expression of marker genes of the Wnt/β-catenin pathway in the AP-AR42J cells was dramatically up-regulated by transfection with PTEN compared with that treated by scramble-shRNA or pCDH. In contrast, these genes in cells transfected by PTEN shRNA exhibited the lowest level as demonstrated by the qualitative and quantitative results. These results together suggest that overexpression of PTEN could signally activate the Wnt/β-catenin pathway.

### Down regulation of inflammatory factors by PTEN

**Fig. 6:**
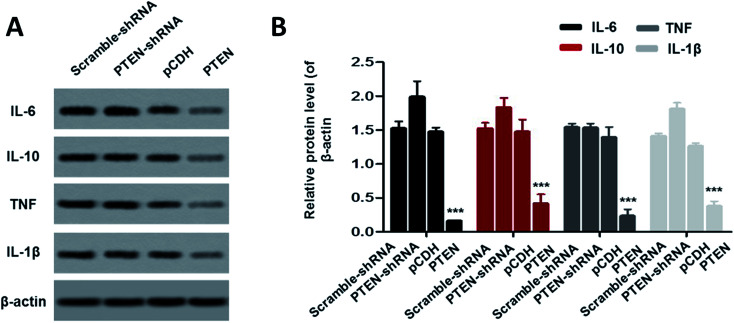
To evaluate the effect of PTEN on alleviating acute pancreatitis induced inflammation, the levels of the inflammatory factors including IL-6, IL-10, TNF and IL-1β were further determined. The western-blot assay (A) exhibited that cells treated by PTEN displayed significant lower levels of those factors compared with the control group, suggesting that over expression of PTEN can obviously restrain the inflammation of acute pancreatitis. Such results were further confirmed by the quantitative analysis (B). Importantly, the experiments were conducted repeatedly three times with ***P < 0.01 being significantly higher than the control.

To investigate the effect of PTEN on alleviating acute pancreatitis induced inflammation, inflammatory factors including IL-6, IL-10, TNF and IL-1β were determined by western-blot assay. As clearly illustrated in Fig. 6A and B, the cells treated by PTEN displayed significantly lower levels of inflammatory factors compared with the scramble-shRNA group and pCDH treated ones. However, after the PTEN genes in the AP model cells were knocked down by the corresponding shRNA, the expression levels of IL-6, IL-10, TNF and IL-1β were remarkably elevated. Taking these results together, we can draw the conclusion that over expression of PTEN can obviously restrain the inflammation of acute pancreatitis.

## Discussion

Acute pancreatitis, one of the commonly diagnosed clinical diseases, has a high incidence and rapid development and progression.^1,2^ Not only limited to pancreas inflammation, there is accumulating evidence demonstrating that the symptoms of AP also include hypotension, insufficient organ perfusion, adult respiratory distress syndrome and multiple organ dysfunction syndrome.^23^ PTEN is generally considered as a tumor suppressor and insufficient expression of PTEN is closely correlated with the occurrence and progress of multiple cancers.^24–26^ Although high levels of PTEN can result in a decreasing lifetime risk of pancreatic cancer,^27–29^ the effect and the underlying mechanisms of PTEN on AP remain elusive.

PI3K/AKT signal, the classical PTEN downstream targets, is essential for maintaining cell growth, survival, death and metabolism of many cancer types.^30^ Previous studies have demonstrated that PTEN holds great potential in negatively regulating the activation of the PI3K/AKT signaling pathway and consequently exerting an inhibitory effect against tumors or alleviating inflammation.^31^ However, in the present study, we speculate that the PI3K/AKT signal pathway is not the only pathway that can be regulated by PTEN and leads to alleviation of disease. The Wnt/β-catenin signaling pathway has been recently recognized to be one of the most important pathways that can cause significant adverse effect on maintaining body functions once it is dysregulated.^32^ Additionally, the role of the Wnt/β-catenin pathway in regulating pancreatic biology, especially pancreatitis, has been illustrated and has attracted much attention.^20,33^ Interestingly, in the present study, the Wnt/β-catenin pathway can be significantly activated by PTEN, as demonstrated by the up-regulation of Wnt3a, β-catenin, TCF4, c-Myc and cyclin D1 in caerulein-induced AR42J cells and pancreatic tissues of rats. By activation of the Wnt/β-catenin signal, acute pancreatitis was significantly alleviated, as is evident by the down-regulated inflammatory factors including IL-6/10, TNF, and IL-1β.

A high-dose of caerulein, the isozymes of cholecystokinin (CKK) and the regulator of pancreatic secretion, have been demonstrated to inhibit the formation of dense mature vacuoles in acinar cells.^33^ However, the disruption of vacuoles can lead to abnormalities in the levels of digestive enzymes and lysosomal hydrolases, which are essential in the activation of intracellular cathepsin B and trypsin.^34^ Importantly, cathepsin B and trypsin are the main causes of the self-digestion of the pancreas.^34^ In this case, we applied caerulein as the inducer to build the rat model of acute pancreatitis. To ensure that the developed rat model and cell model of acute pancreatitis possess consistent pathogenesis and pathophysiologies, the cell model of acute pancreatitis has been developed by the same inducer as the animal models. An obvious signal of PTEN was observed in the tissues of AP models but was not detectable in the normal ones, confirming the successful establishment of AP rats.

As reported previously, PTEN can serve as a crucial modulator of the migration and invasion of various cancer cell types.^35^ However, the impact of PTEN on the migration and invasion of AP cells is still unclear. Wound-healing experiments performed here displayed that the cells treated by PTEN have the widest gap and the minimum crystal violet compared with others. In contrast, after transfection with PTEN-shRNA, migration and invasion ability was immediately recovered while the control genes scramble-shRNA and pCDH have no effect. Transwell assays were further conducted and cells incubated with PTEN-shRNA showed the most amount of crystal violet, indicating an active invasion was obtained by the PTEN-unrestrained AP cells. However, such behavior was significantly depressed after incubating the cells with sufficient PTEN.

Although there is direct evidence of PTEN suppressing the growth, migration, and invasion of AP cells, the underlying mechanisms are still unclear. It is widely accepted that the Wnt/β-catenin pathway activation has a significant inhibition effect on the migration and invasion of various cancer cells.^36–38^ Based on this, the correlations between the Wnt/β-catenin pathway and the migration and invasion of AP cells are required to be investigated in detail. Interestingly, the cells treated by PTEN and displaying incompetent wound-healing ability were highly positive to the downstream genes of Wnt/β-catenin pathway including Wnt3a, β-catenin, TCF-4, cMyc, and cyclin D1. However, silencing PTEN genes with the corresponding shRNA led to the strongest capacity of migration and invasion of AP cells and decreased levels of the genes mentioned above. Collectively, we can draw the conclusion that the migration and invasion of acute pancreatitis was suppressed by PTEN through activating the Wnt/β-catenin pathway.

As the role and mechanism of PTEN in suppressing AP cells growth, migration, and invasion has been thoroughly analyzed, PTEN was therefore supposed to hold great potential in alleviating AP inflammation. For verification, appropriate detection indexes are crucial. TNF, the most important inflammatory mediator, increases the permeability of vascular endothelial cells by activation of neutrophils and lymphocytes, regulates the metabolic activity of organs and promotes the synthesis and release of other cytokines.^39^ In addition, other inflammatory factors such as IL-6, IL-10 and IL-1β are closely related to histrionic necrosis, blood flow congestion and multi-organ dysfunction.^40,41^ As demonstrated, high levels of the above inflammatory factors in AP model cells could be significantly decreased by treating with PTEN. In contrast, with post transfection with PTEN-shRNA, an obvious up-regulated expression of IL-6, IL-10, TNF and IL-1β was detectable. Based on these, the PTEN could be confirmed to be an efficient alleviator for the inflammation of acute pancreatitis.

In conclusion, the results of the present study demonstrate the excellent restraining effect of PTEN on the inflammation, viability and motility of acute pancreatitis. Through activating the Wnt/β-catenin pathway, the expression of PTEN regulates the levels of Wnt3a, β-catenin, TCF-4, c-Myc, and cyclin D1 to inhibit the viability and motility of AP cells. Besides, the effect of PTEN on the alleviation of inflammation was achieved by down-regulation of relevant cytokines including IL-6, IL-10, TNF and IL-1β. Taken together, the present work has indicated the great potential of PTEN in the treatment of acute pancreatitis. Furthermore, although chronic pancreatitis was not studied here, previous studies have demonstrated that the PTEN expression in acute pancreatitis d and chronic pancreatitis was uniformly intact,^42^ suggesting that PTEN also holds great potential in the treatment of chronic pancreatitis.

## Conflicts of interest

The authors declare no competing financial interests.

## Abbreviations

AP: Acute pancreatitis
PTEN: Phosphate and tension homology deleted on chromosome ten
SD: Sprague-Dawley
ELISA: Enzyme-linked immune sorbent
qRT-PCR: Real-time reverse transcription PCR
IL: Interleukin
TNF: Tumor necrosis factor

## Supplementary Material
